# Reading Wishes from the Lips: Cancer Patients’ Need for Psycho-Oncological Support during Inpatient and Outpatient Treatment

**DOI:** 10.3390/diagnostics12102440

**Published:** 2022-10-09

**Authors:** Jan Ben Schulze, Marc Dörner, Hermanas Usas, Moritz Philipp Günther, Roland von Känel, Sebastian Euler

**Affiliations:** Department of Consultation-Liaison-Psychiatry and Psychosomatic Medicine, University Hospital Zurich, University of Zurich, 8091 Zurich, Switzerland

**Keywords:** artificial neural network (ANN), back propagation neural network (BPNN), wish for psycho-oncological support, distress, psycho-oncology, cancer

## Abstract

Background: Psycho-oncological support (PO) is an effective measure to reduce distress and improve the quality of life in patients with cancer. Currently, there are only a few studies investigating the (expressed) wish for PO. The aim of this study was to evaluate the number of patients who request PO and to identify predictors for the wish for PO. Methods: Data from 3063 cancer patients who had been diagnosed and treated at a Comprehensive Cancer Center between 2011 and 2019 were analyzed retrospectively. Potential predictors for the wish for PO were identified using logistic regression. As a novelty, a Back Propagation Neural Network (BPNN) was applied to establish a prediction model for the wish for PO. Results: In total, 1752 patients (57.19%) had a distress score above the cut-off and 14.59% expressed the wish for PO. Patients’ requests for pastoral care (OR = 13.1) and social services support (OR = 5.4) were the strongest predictors of the wish for PO. Patients of the female sex or who had a current psychiatric diagnosis, opioid treatment and malignant neoplasms of the skin and the hematopoietic system also predicted the wish for PO, while malignant neoplasms of digestive organs and older age negatively predicted the wish for PO. These nine significant predictors were used as input variables for the BPNN model. BPNN computations indicated that a three-layer network with eight neurons in the hidden layer is the most precise prediction model. Discussion: Our results suggest that the identification of predictors for the wish for PO might foster PO referrals and help cancer patients reduce barriers to expressing their wish for PO. Furthermore, the final BPNN prediction model demonstrates a high level of discrimination and might be easily implemented in the hospital information system.

## 1. Introduction

Cancer patients have to endure a high burden of distress [1]. Distress in cancer patients is defined as a “multifactorial unpleasant experience of psychological (i.e., cognitive, behavioral, emotional), social, spiritual, and/ or physical nature that may interfere with the ability to cope effectively with cancer, its physical symptoms, and its treatment” [2]. When being assessed, 25% to 60% of cancer patients report distress [3,4,5]. Research indicates that high levels of distress negatively affect the quality of life (QoL) [6,7], patient satisfaction with treatment [8,9] and treatment adherence [10,11]. Furthermore, cancer patients with high psychological distress tend to have more problems coping with their disease [7] and are more susceptible to disability in the course of the disease [12,13]. Prior studies emphasized that psycho-oncological support (PO) is an effective measure to improve patients’ QoL and reduce distress in patients with cancer [7,14,15,16]. Moreover, complementary PO has a positive effect on cost–utility ratios and total healthcare costs are lower if patients ask for PO [17].

Usually, PO includes a broad range of services, such as cost-free counseling centers close to home and external or internal psychiatric respectively psychological consultations [18]. There are two psychological dimensions of cancer that PO is concerned with. First, the psychosocial dimension, which comprises the emotional responses of patients at all stages of the disease, their families and caretakers. Second, the psychobiological dimension, including the psychological, behavioral and social factors that may influence cancer morbidity and mortality [19]. 

In a larger context, PO represents various aspects of social support, which can be divided into social-emotional (relationships rich in affection), instrumental (based on advice and practical suggestions), problem-oriented (focused on the resolution of a specific problem) and daily support [20,21]. Perceived positive social support is important to help people adjust to the disease by decreasing distress. However, there are also unsupportive social relationships, which should be taken into account. Unsupportive social support cannot only be reduced to the lack of support but further implies social interactions that do not fit the needs of cancer patients. Hence, caregivers must be aware that in some cases their support might be unintentionally perceived as negative, depending on many factors, such as patients’ personality traits and cognitive representations, the larger social and cultural context as well as on the specific interventions provided by the caregiver [21]. Sebri et al. recently pointed out that structured psychological interventions based on the training of positive social support are essential to prevent the mental burden caused by cancer and to better cope with the so-called “Injured Self”. The Injured Self is a term describing how a chronic disease may deeply affect the self-representation of patients regarding their body and identity [21,22]. Several works demonstrated the reciprocal interconnection between self-representations and autobiographical memories as a patient and the negative consequences of an Injured Self, especially in patients with breast cancer [21,22,23,24]. Despite the importance of adequate PO, a considerable amount of distressed cancer patients lacks professional help [14,25,26] for which there are multiple reasons. Referrals to PO can be increased by routine distress screening [27]. However, there are still many barriers to screening for distress in patients with cancer. These barriers include patients with specific types of cancer, such as breast or skin cancer, inpatient treatment of less than 28 days, patients with a mental disorder and psychotropic medication and those whose disease is not discussed at a tumor board [28,29]. Other barriers may be a lack of hospital staff, lack of time and lack of training of caregivers [30,31]. 

As well as these barriers to PO for patients with cancer, there have been some studies about factors, which predict the wish for PO. For instance, low emotional wellbeing, lack of social support, younger age, higher level of education, emotional burden unrelated to the disease, lung and breast cancer [18], current or previous depressive mood [27], high level of anxiety [32] and living in a rural area [33] predicted the wish for PO. However, these studies included small sample sizes, only specific types of cancer or patients aged 60 years or older.

Finally, different healthcare systems across the globe may have some influence on the wish for PO. Although there have been many improvements in providing PO for patients with cancer during the last decades [19], financial aspects have to be considered. For instance, in the United States (U.S.), patients are often required to pay for services. Thus, they may not engage in support services even if those are offered. Dee et al. highlighted a growing proportion of cancer patients in the U.S. who experience financial worries and distress due to healthcare costs [34]. On the other hand, in many European countries, such as Germany and Switzerland, healthcare costs (including PO) are almost completely covered by insurance. 

Our study aimed (1) to evaluate the number of patients who wish for PO and (2) to identify predictors for the wish for PO in a large patient population with all types of cancer. As a novelty, this study also aimed (3) to build a prediction model for the wish for PO in patients with cancer based on the Back Propagation Neural Network (BPNN). 

Previous research is based on conventional statistical methods, such as logistic regression analysis. However, these statistical procedures cannot fully represent the multifactorial and intertwined causes for the wish of PO in patients with cancer, and thus, tend to be inaccurate. On the other hand, BPNN is based on the Artificial Neural Network (ANN), which simulates the structure and function of the human brain. Being an adaptive, self-learning and self-organizing system, ANN is able to identify complex non-linear relationships between variables [35]. Moreover, BPNN has already proven its usefulness regarding several psychiatric and psychosomatic investigations in the past [36,37,38,39,40,41]. Overall, BPNN is better suited to determine individual prediction accuracy, and thus, might help increase the uptake of PO [42].

## 2. Methods

### 2.1. Subjects and Data Collection

**Figure 1:**
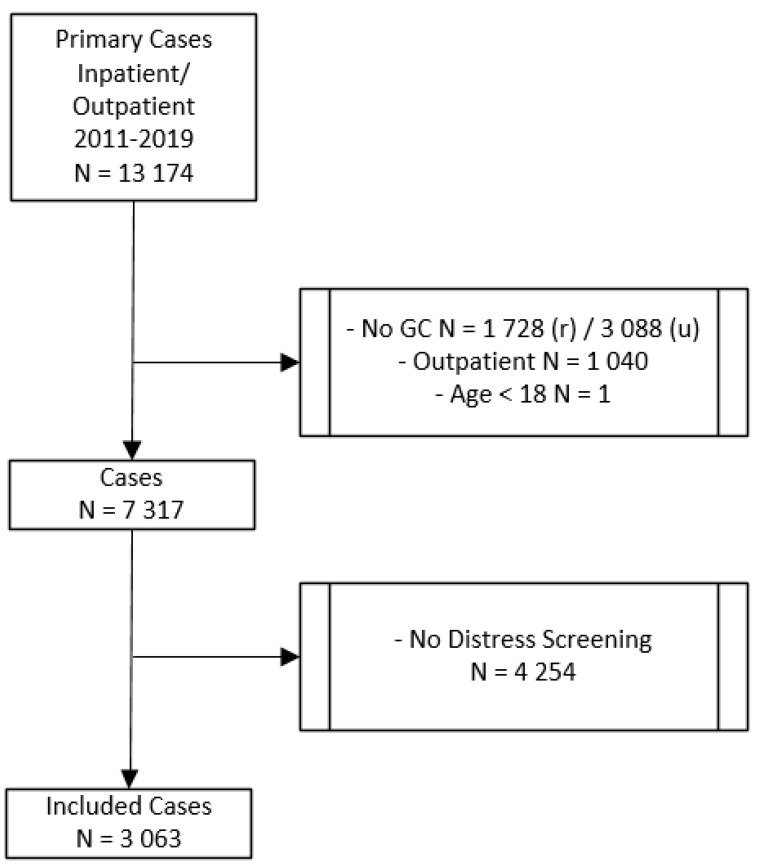
Procedure of selecting the study sample. GC = general consent; r = rejected; u = unkown; outpatient = treated as outpatients only.

In this study, we retrospectively included 3063 patients with an initial diagnosis of cancer who were diagnosed and treated at the Comprehensive Cancer Center Zurich (CCCZ) at the University Hospital Zurich. Data were collected from the case files of cancer in- and outpatients between 2011 and 2019. The procedure of selecting the study sample is depicted in Figure 1. There were 13,174 total cases with an initial diagnosis of cancer. All patients diagnosed with cancer before 2011 were excluded from the study. Only those patients were included in the study, who were diagnosed for the first time with cancer. The final study sample included patients who signed a general consent for research at the beginning of their treatment, who were treated either as inpatients or as both in- and outpatients, who were older than 18 years, and who were screened for distress and asked if they need PO. If patients were asked more than once, only the first distress screening was included in our analysis. The study was approved by the Ethics Committee of the State of Zurich, Switzerland (BASEC NR. 2020-00977; June 2020).

The data in our study represent all types of cancer. The proportions of the type of cancer are illustrated in Table 1. 

### 2.2. Measurements

Distress, as defined in the introduction, was assessed with the distress thermometer (DT), which is a commonly used screening tool. Cancer nurses are requested to administer the DT repeatedly during inpatient treatment and outpatient visits in patients with cancer [43]. The DT is a visual 11-point scale, ranging from 0 (no distress) to 10 (highest level of distress). Previous studies suggested a DT cut-off score of 5 or higher as an indicator of a potential referral to PO [28,44]. Patients who are screened for distress are simultaneously asked by nurses if they have a wish for PO, pastoral care or social services. Patients who score above the cut-off and/or wish for PO, are referred to internal PO services of the cancer center by the treating physician. Subsequently, a psychiatrist or psychologist at the hospital provides supportive interventions to help patients better cope with their disease. Then, together they decide if further psychotherapy is necessary.

### 2.3. Statistical Procedures

We used IBM SPSS Statistics, version 27 (Chicago, IL, USA) for statistical analysis and Matlab (R2019a. Mathworks Inc., Natick, MA, USA) for BPNN computations. Mean scores, standard deviation and relative and absolute distributions were calculated to describe the prevalence of the wish for PO, distress scores and patient characteristics. An unpaired t test was conducted to compare differences between the number of patients who wish for PO and distress scores. To identify factors that predict the wish for PO, we performed a binary logistic regression analysis with the wish for PO (yes/no) as the dependent variable. In the first step, we analyzed associations of sociodemographics (see also Table 2) and other variables, such as type of cancer, the cancer stage at first diagnosis, current psychiatric diagnoses, opioids and non-opioid analgesics administered during treatment, patients’ request for pastoral care and social services support. The stage of cancer was classified according to the Union for International Cancer Control (UICC) [45]. Moreover, we tested the age-adjusted Charlson comorbidity index (CCI) as a potential predictor for the wish for PO. The CCI has been developed to predict the contribution of chronic comorbid diseases to mortality [46,47,48]. Using the backward elimination method, significant variables of each data block were chosen and entered into the final model. Most of the analyzed variables were dichotomized as “0 = No” and “1 = Yes”, except for age (metric scale) and nationality (categorical variable: Switzerland, Europe (including Switzerland) and non-European). Significance level (two-sided *p*-value) was set at *p* < 0.05. 

In a BPNN, information migrates from input layers through hidden layers to output layers. The information in this network only moves in one direction. Hence it is called a feedforward network. To minimize differences between the final calculated output and the target output from the training data, iterations are made based on the error rate obtained in the previous run. By reducing error rates, the BPNN model will become more reliable and generalizable. In this way, BPNN is a standard method of training and adaptively controlling artificial neural networks [35,38].

## 3. Results

### 3.1. Description of the Study Sample

Table 2 demonstrates sociodemographic and clinical variables of the final study sample. Two-thirds of the study sample were male participants. The average age (± SD) was 61.5 ± 13.9 years (range 18–95). 

### 3.2. Prevalence of the Wish for PO

The absolute and relative distribution of the wish for PO and the associated distress scores are illustrated in Table 3. In total, 1752 patients (57.19%) had a distress score above the cut-off. Of those, only 20.89% wished for PO. A total of 81 patients (2.64%) below the cut-off also wished for PO. Of all included cancer patients, 14.59% expressed the wish for PO. Thus, the difference between the quantity of distress scores of five or higher and the prevalence of the wish for PO is significant (t = 63.96, df = 3062, *p* < 0.001). Regarding sex differences, women wished more often for PO and were more frequently distressed than men.

### 3.3. Identification of Predictors for the Wish for PO

**Figure 2:**
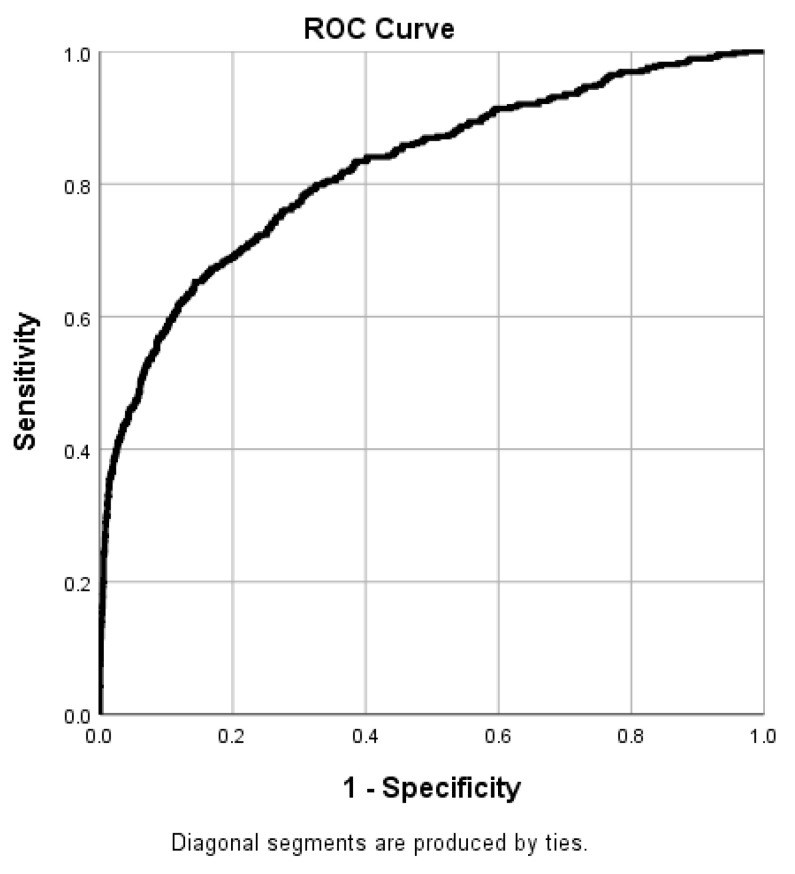
Receiver operating characteristic (ROC) curve of the binary logistic regression model (area under curve (AUC): 0.822).

Figure 2 displays the receiver operating characteristic (ROC) curve of the binary logistic regression model. The chi-square test revealed that the logistic regression model was significant (*X*^2^ = 1864.462, *p* < 0.001). The model explained 36.0% of the variance (Nagelkerke R^2^) and correctly classified 82.2% of cases, indicating high model quality. The model had a sensitivity of 37.8% and a specificity of 98%.

Table 4 highlights the nine independent variables in the significant equation from the binary multivariable logistic regression analysis. There was no evidence of multicollinearity. Collinearity statistics revealed that the variance inflation factor (VIF) and tolerance for each significant variable were below, respectively, and above the values suggested in literature (VIF <10 and tolerance >0.1; [49]). Patients who wished for pastoral care or social services demonstrated the highest odds ratios (OR= 13.1 and 5.4). The third most significant predictor for the wish for PO was sex. Females had 1.8-times higher odds to wish for PO than males. Opioid treatment and a current psychiatric disorder also predicted the wish for PO. Compared to other cancer types, patients with skin cancer and hemato-oncological malignancies were more likely to ask for PO, while malignant neoplasms of digestive organs and older age negatively predicted the wish for PO. All other examined cancer types, stage of cancer at first diagnosis, non-opioid analgesics, CCI and other sociodemographic variables, such as marital status, nationality and native tongue did not predict the wish for PO.

### 3.4. BP Neural Network Structure

**Figure 3:**
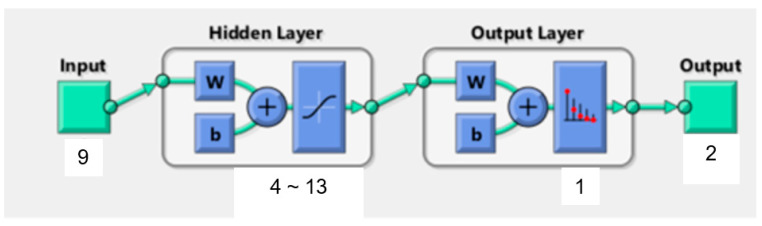
Structure of BP neural network. w: weights coefficient (w). b: threshold value (b).

In the current study, the BPNN consisted of one input layer, one hidden layer and one output layer. Prior studies already demonstrated that this typical three-layer BPNN is a reasonable compromise between model accuracy and network complexity [41]. Figure 3 illustrates the structure of the BPNN model for patients’ wishes for PO. The nine significant independent variables determined by logistic regression analysis represent the number of input neurons. The output neurons are defined by the dichotomous dependent variable wish for PO (0 = no, 1 = yes). We used the equation H = √M + N + α to calculate the number of neurons in the hidden layer. M depicts the number of output neurons, N is the number of input neurons and α a constant from one to ten.

### 3.5. Training of the BP Neural Network

Matlab randomly divided the data into a training sample (70%), a validation sample and a test sample (15% each). In our BPNN model for patients’ wishes for PO, the hidden layer neurons are in a range between four and 13. Theoretically, a higher coincidence rate (π) and a lower number of iterations lead to a higher BPNN model accuracy. A different number of hidden layer neurons results in different evaluation indexes (sensitivity, specificity, positive predictive value, negative predictive value, coincidence rate (π)) and varying numbers of iterations. Thus, BP neural network training and repetitious data simulation processes were carried out under different numbers of hidden layer neurons. Matlab indicated that the highest BPNN model accuracy was achieved in a range between seven and nine neurons (see Table 5). 

Another issue of the BPNN training is that different values of the weights coefficient (w) and the threshold value (b) lead to different learning process models. Since the initial value of (w) and (b) is a random number between −1 and 1, repetitious data simulation processes with different initial values of (w) and (b) and a number of hidden layer neurons from seven to nine were performed via Matlab. Comprehensively considering the evaluation indexes and the number of iterations, Network 3 with eight neurons in the hidden layer presents the optimal BPNN model for patients’ wishes for PO (Table 6). It has a total sensitivity of 31.2%, a specificity of 98.8%, a positive predictive value of 82.0%, a negative predictive value of 89.2%, a total coincidence rate (π) of 88.8% and 13 iterations. For further details regarding the computer program of the optimal BPNN model with eight hidden layers neurons, see Appendix A.

### 3.6. Validation of the BP Neural Network

**Figure 4:**
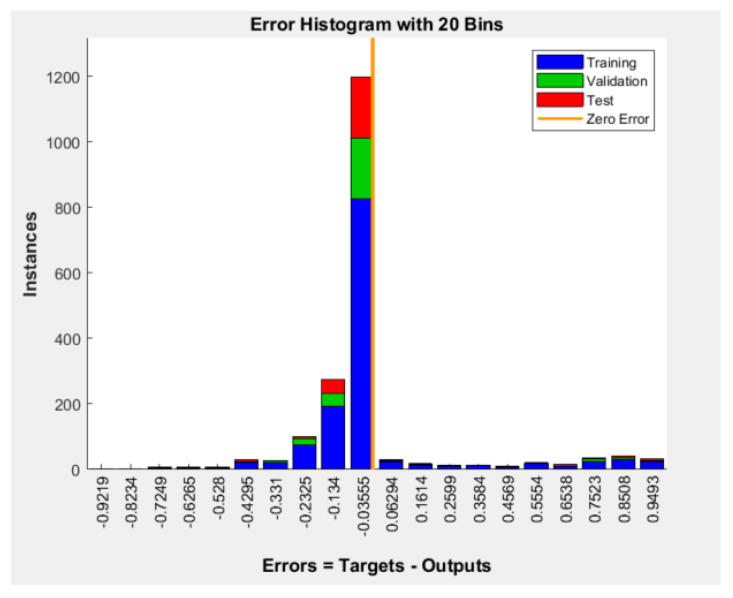
Error histogram of the BPNN model for patients’ wishes for PO.

Figure 4 shows the error histogram of the BPNN model (errors = targets-outputs). Most of the model’s errors are concentrated around the zero error line, which indicates predominantly small errors.

**Figure 5:**
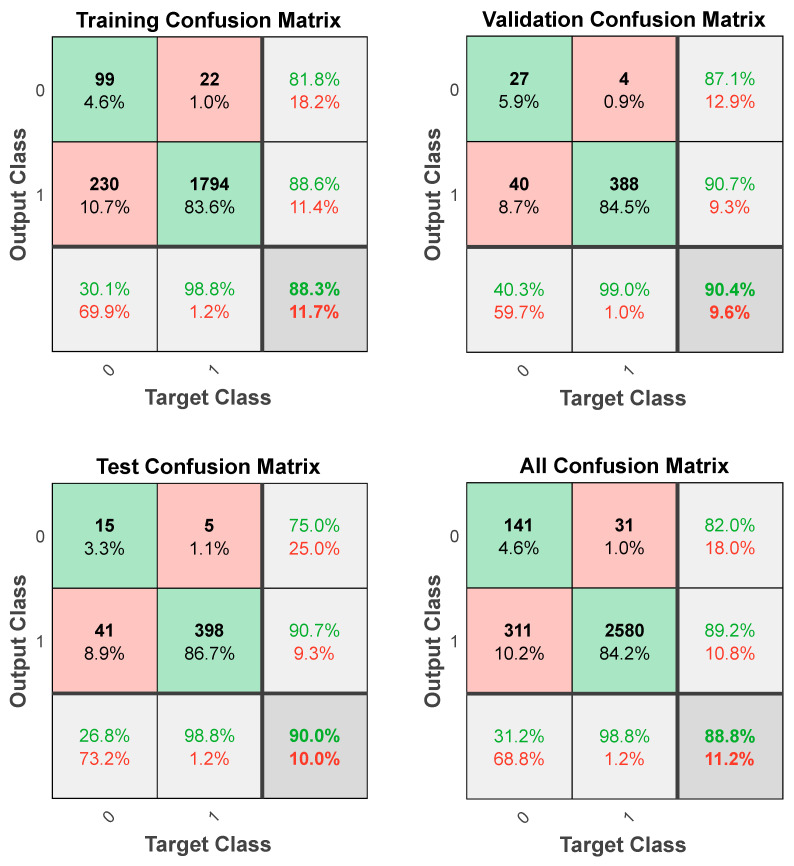
Confusion matrices of the BPNN model.

**Figure 6:**
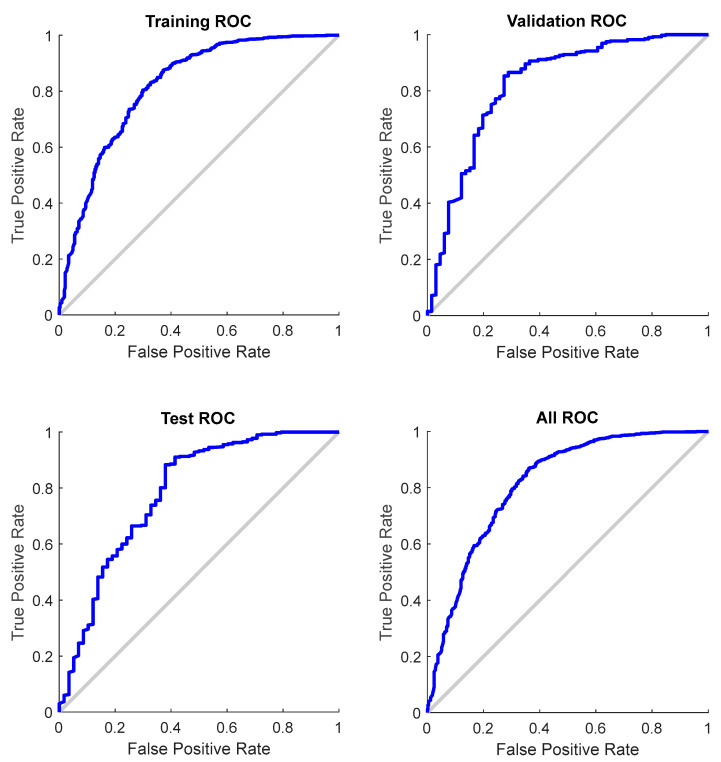
ROC curve for each sample of the BPNN model (total AUC: 0.816).

The confusion matrices demonstrate a high coincidence rate (π) for each sample (Figure 5; bottom right corner). Figure 6 depicts the ROC curve of the BPNN model, which correctly discriminated 81.6% of all cases. In total, this suggests a high-BPNN model quality with outstanding discrimination efficiency.

## 4. Discussion

This study investigated the number of cancer patients with a wish for PO during inpatient and outpatient treatment in a naturalistic setting of a comprehensive cancer center and identified predictors for the wish for PO. As a novelty, this study further utilized BP neural network analysis to build a precise prediction model for patients’ wishes for PO, and thus, aimed to improve individual prediction accuracy.

In this study, 57.19% of cancer patients were identified as distressed and 14.59% wished for PO. These findings align with prior studies (U.S., Germany, Asia) in clinical settings that explored incidence rates of distress and the wish for PO in patients with cancer [3,4,5,18]. However, the conclusions of previous studies (Germany) in controlled and experimental settings were based on significantly lower rates of reported distress and wish for PO [50,51]. This underlines the relevance of naturalistic data in evaluating distress levels and the wish for PO in cancer patients. 

The large gap between reported distress and the wish for PO is of note. A possible explanation for this discrepancy might be a phenomenon characterized as a “normality paradox” [52]. Some patients decline PO to sustain a sense of normality in everyday life. Additionally, some cancer patients assess their distress levels as not severe enough for treatment, and hence, are not convinced that PO is necessary (Australian study) [53]. 

Moreover, the cultural and medical context of PO involving different healthcare systems, treatment regimens and lifestyles in the U.S., Europe, Asia and across the globe should also be considered [19,34]. Perceived stigma in older individuals [54,55], male sex [56] and certain cultures [57] might be a barrier to PO and psychotherapeutic support in general (studies in the U.S. and Europe). Destigmatization of psychosocial problems [27] and providing cancer patients with adequate information about the importance of PO could help in reducing these barriers (studies across the globe) [58,59]. The existing literature suggests that especially oncologists who skillfully recommend PO might improve patients’ uptake of those services (Swiss study) [60]. Additionally, the identification of specific subgroups of cancer patients with different distress profiles might contribute to a more tailored treatment offer (Swiss study) [61] reducing the gap between distress and the request for PO.

In our study, we identified nine predictors that each independently influenced the wish for PO. Younger age (in line with a prior German study [18]) and female sex (contrary to other studies) predicted the wish for PO, which might be due to the already mentioned perceived stigma by older persons [54,55] and male sex [56]. Prior studies pointed out that younger age and female sex are also predictive of higher levels of distress (U.S. and Korean studies; [62,63,64,65]). A current psychiatric disorder was additionally predictive of the wish for PO. This finding is in accordance with an Austrian study [27]. The barrier to accepting PO could have been lower for those patients because they were more likely to have experienced psychiatric treatment before, and thus, might be more open to this kind of therapeutic option. However, there are studies suggesting that patients with a psychiatric disorder are rather neglected by healthcare professionals and less likely to be screened for distress and to receive PO (Swiss studies) [29,66]. Consequently, patients with cancer and a psychiatric diagnosis might be a vulnerable subgroup, which needs further clinical and research attention. Regarding medication, opioid treatment was predictive of the wish for PO. In line with this result, Günther et al. [66] indicated that patients are more likely to be screened for distress and receive PO when being treated with opioids in a previous study. Patients with opioid treatment might wish more often for PO due to possible side effects of the medication and higher pain levels. Regarding the specific types of cancer, skin cancer and hemato-oncological malignancies predicted the wish for PO, while malignant neoplasms of digestive organs were predictive of an absent wish for PO. Other studies pointed out that patients with hemato-oncological malignancies report the highest levels of distress [18], and malignant neoplasms of the skin, especially melanoma, are difficult to treat and also cause high levels of distress due to poor survival rates (U.S. study) [67]. This might be an explanation for the predictive wish for PO in those patients.

Interestingly, a new finding of our study is that patients’ requests for pastoral care and social services support predicted the wish for PO and additionally demonstrated the highest odds ratios (OR= 13.1 and 5.4). It is not surprising that distressed patients in need of psychosocial support request all the help they are offered. We also assume that patients might struggle to differentiate between the specific value of the different entities. On the other hand, PO, social services and pastoral care can be highly synergistic in reducing patients’ distress. For instance, social services reduced distress in patients with cancer significantly (Australian study) [68]. Cancer patients who are in contact with social services also seem to agree more in accessing and approaching further support services and feel more supported in coping with strong emotions and managing challenges related to cancer [68]. Other studies (U.S.) emphasized the significance of pastoral care, especially for patients with advanced stages of cancer [69] in defining their purpose in life and accepting their illness [70]. In consequence, the three disciplines might reduce distress in cancer patients significantly through interprofessional collaboration (Asian study) [71]. However, given the limitation of resources, a more detailed explanation by cancer nurses about the kind of assistance each service provides could be helpful for a more specific referral to the respective psychosocial support service. 

As a novelty, we established a prediction model for patients’ wishes for PO based on BPNN including all nine significant variables determined by logistic regression. In sum, it demonstrates a high level of discrimination, which is comparable to our regression analysis model. Although the established BPNN model is of clinical significance, it cannot reach its full potential due to the relatively low number of independent variables. However, the present study provides evidence that BPNN is able to accurately manage real-world data. Compared to conventional methods, BPNN has the advantage of better individual prediction accuracy and precise data fitting based on its adaptability and simulation of the human brain. Moreover, BPNN is better suited to handle a large sample with different participants’ characteristics due to its ability to identify complex non-linear relationships between variables [35,36,37,38,39,40,41,42]. Combining both methods can facilitate the identification of predictors for the wish for PO. 

Strengths and Limitations

Our study has several strengths, such as the large study sample, including all types of cancer and treatment options and utilizing advanced statistical approaches. Still, there are some notable limitations. Our study utilized data from a single CCC in Switzerland, which limits generalizability. However, the quality of the data and of the obtained results is high, because the documentation of patient-related information in the present study followed strict institutional standards. An important limitation is the fact that some types of cancer, such as gynecological, urological and endocrinological malignant neoplasms, were underrepresented in the current study (see Table 1). Günther et al. [29] assumed interinstitutional differences to be responsible for low screening rates for certain cancer types. A selection bias is the inclusion of only initial diagnoses of cancer, which allowed the exclusion of confounders. The majority of our patients were male, limiting the representativeness of the sample. Prospective studies should strive to include more female patients, because social support, including PO, may be strongly perceived as different, as we already highlighted in the introduction [20,21,22]. We did not compare distress levels and the wish for PO in different cancers although research has shown that patients with different cancers have different needs for PO [18]. There are some reasons why we did not include distress as a potential predictor for the wish for PO in our regression analysis, which might be a limitation of our study. First, previous studies (U.S. and Europe) suggested different DT cut-off scores for specific cancer patient populations [72,73]. The study was carried out in a hospital, where only one specific distress cut-off score is used. Second, we kept in mind the already mentioned large gap between reported distress and the wish for PO in prior studies [3,4,5,18]. Another limitation is that the DT is a single-item screening tool, and thus, may not adequately measure distress. The use of additional screening tools, such as the Hornheide Screening Instrument [18], should be considered in future studies. To improve clinical care for patients, further exploration of reasons for acceptance or rejection of PO in specific cancer types is needed (Swiss study) [74]. Finally, there is no standard procedure for constructing the BP neural network. 

## 5. Conclusions

The present study highlights a discrepancy between patients with a distress score above the cut-off value and those that wish for PO. With this study, we identified novel predictors for the wish for PO. Considering these factors could help increase PO utilization, thereby reducing distress and improving QoL in patients with cancer. Improved discrimination between the content of PO, social services and pastoral care as well as strengthening interprofessional collaborations might help to better meet patients’ individual needs. Future studies could also compare the percentage of patients who expressed the wish for PO with those who actually received it and investigate whether patients are involved in psychotherapy sessions. Patients might not be fully aware of all possible multifactorial causes for their wish for PO. BPNN is able to identify those complex relationships between variables that otherwise might have been overlooked. Moreover, the implementation of BPNN prediction algorithms in the hospital information system might help identify patients who need PO more easily. 

## Figures and Tables

**Table 1:** Type of cancer in patients studied.

ICD-10 (3-Digit Diagnosis Code)	Type of Cancer	2011–2019	(%)
		N	
C00-C14	Malignant neoplasms of lip, oral cavity and pharynx	423	13.8
C15-C26	Malignant neoplasms of digestive organs	345	11.3
C30-C39	Malignant neoplasms of respiratory and intrathoracic organs	542	17.7
C40-C41	Malignant neoplasms of bone and articular cartilage	3	0.1
C43	Melanoma and other malignant neoplasms of skin (without basalioma)	135	4.4
C45-C49	Malignant neoplasms of mesothelial and soft tissue	44	1.4
C50	Malignant neoplasms of breast	30	1
C51-C58	Malignant neoplasms of female genital organs	42	1.4
C60-C63	Malignant neoplasms of male genital organs	384	12.5
C64-C68	Malignant neoplasms of urinary tract	104	3.4
C69-C72	Malignant neoplasms of eye, brain and other parts of central nervous system	374	12.2
C73-C75	Malignant neoplasms of thyroid and other endocrine glands	37	1.2
C76-C80	Malignant neoplasms of ill-defined, other secondary and unspecified sites	4	0.1
C81-C96	Malignant neoplasms of lymphoid, hematopoietic and related tissue	586	19.1
D00-D09	In situ neoplasms	10	0.3
**Total**		**3063**	**100**

Note: ICD-10: International Statistical Classification of Diseases and Related Health Problems, Version 10. N: number.

**Table 2:** Description of the study sample.

	Total	Women	Men
Patients	**N (%)**	1022 (33.3%)	2041 (66.6%)
	3063 (100)		
Age (mean and SD; range)	61.5 (13.9); 18–95	60.6 (14.8); 18–92	62 (13.4); 18–95
Married	1750 (57%)	511 (50%)	1239 (60.7%)
Primary language German	3019 (98.6%)	1012 (99%)	2007 (98.3%)
Nationality			
ch	2516 (82.1%)	863 (84.4%)	1653 (81.0%)
eu (non-ch)	396 (12.9%)	114 (11.1%)	282 (13.8%)
non-eu	151 (4.9%)	46 (4.5%)	105 (5.1%)
Advanced stage of cancer	1451 (47.4%)	526 (51.5%)	925 (45.3%)
CCI (≥ 5)	1865 (61.4%)	625 (61.2%)	1240 (60.7%)
Distress score ≥ 5	1752 (57.1%)	675 (66%)	1085 (53.1%)
Psychiatric diagnosis present	209 (6.8%)	72 (7%)	137 (6.7%)

Note: N: number. SD: standard deviation. ch: Switzerland, eu: Europe, non-eu: non-European. CCI: Age-adjusted Charlson comorbidity index.

**Table 3:** Absolute and relative distribution of the wish for PO and associated distress scores.

		Total	Men	Women
		**N (%)**		
Wish for PO	Distress score ≥ 5	366 (11.94)	184 (9.01)	182 (17.79)
Wish for PO	Distress score < 5	81 (2.64)	48 (2.35)	33 (3.22)
No wish for PO	Distress score ≥ 5	1386 (45.24)	896 (43.92)	490 (47.89)
No wish for PO	Distress score < 5	1230 (40.15)	912 (44.70)	318 (31.08)
	total	3063 (100)	2040 (100)	1023 (100)
	Wish for PO	**447 (14.59)**	232 (11.37)	215 (21.01)
	No wish for PO	2616 (85.40)	1808 (88.62)	808 (78.98)
	Distress score ≥ 5	**1752 (57.19)**	1081 (52.99)	671 (65.59)
	Distress score < 5	1311 (42.80)	960 (47.05)	351 (34.31)

Note: N: number. PO: psycho-oncological support. Significant difference between the number of patients with a distress score of five or higher and the wish for PO is marked bold.

**Table 4:** Binary logistic regression for the wish for PO in patients with cancer.

Variables	Standard B	OR	*p* Value
Request for social services	0.137	5.431	<0.001
Request for pastoral care	0.180	13.094	<0.001
Psychiatric diagnosis present	0.214	1.457	0.078
Opioid treatment	0.130	1.409	0.008
Age	0.004	0.984	<0.001
Female sex	0.123	1.796	<0.001
Malignant neoplasms of digestive organs	0.233	0.584	0.0021
Melanoma and other malignant neoplasms of skin (without basalioma)	0.276	1.720	0.049
Malignant neoplasms of lymphoid, hematopoietic and related tissue	0.144	1.666	<0.001
Nagelkerke R^2^	0.360		

Note: OR: odds ratio.

**Table 5:** Predicted evaluation indexes with a different number of hidden layer neurons.

Nhidden	Se	Sp	1-Sp	PV+	PV-	π	Number of Iterations
7	30.1	98.8	0.012	81.1	89.1	88.6	24
8	31.2	98.8	0.012	82.0	89.2	88.8	13
9	37.6	97.6	0.024	73.3	90.0	88.8	19

Note: Nhidden: Number of hidden layer neurons. Se: Sensitivity. Sp: Specificity. PV+: Positive predictive value. PV-: Negative predictive value. π: Total coincidence rate.

**Table 6:** Predicted evaluation indexes with different initial values of the weights coefficient (w) and the threshold value (b).

		π			Total Sample					Number of Iterations
Network	Nhidden	Training Sample	Verification Sample	Test Sample	Se	Sp	PV+	PV-	π	
1	7	88.8	88.0	88.5	34.7	98.8	74.8	89.7	88.6	28
2	7	88.4	89.5	88.7	30.1	98.8	81.1	89.1	88.6	24
3	8	88.3	90.4	90.0	31.2	98.8	82.0	89.2	88.8	13
4	8	89.1	86.5	88.5	40.7	96.9	69.7	90.4	88.6	18
5	9	88.9	89.3	87.6	37.6	97.6	73.3	90.0	88.8	19
6	9	89.5	89.3	86.5	36.7	98.0	76.5	90.0	89.0	33

Note: Nhidden: Number of hidden layer neurons. Se: Sensitivity. Sp: Specificity. PV+: Positive predictive value. PV-: Negative predictive value. π: Coincidence rate.

## Data Availability

The data presented in this study are available on request from the corresponding author.

## Appendix A

The computer program of the optimal BPNN model with eight hidden layers neurons.

Script Matlab (advanced)

% Solve a Pattern Recognition Problem with a Neural Network

% Script generated by Neural Pattern Recognition app

% Created 02-Apr-2022 11:24:55

%

% This script assumes these variables are defined:

%

% data - input data.

% data_1 - target data.

x = data';

t = data_1';

% Choose a Training Function

% For a list of all training functions type: help nntrain

% 'trainlm' is usually fastest.

% 'trainbr' takes longer but may be better for challenging problems.

% 'trainscg' uses less memory. Suitable in low memory situations.

trainFcn = 'trainscg'; % Scaled conjugate gradient backpropagation.

% Create a Pattern Recognition Network

hiddenLayerSize = 8;

net = patternnet(hiddenLayerSize, trainFcn);

% Choose Input and Output Pre/Post-Processing Functions

% For a list of all processing functions type: help nnprocess

net.input.processFcns = {'removeconstantrows','mapminmax'};

% Setup Division of Data for Training, Validation, Testing

% For a list of all data division functions type: help nndivision

net.divideFcn = 'dividerand'; % Divide data randomly

net.divideMode = 'sample'; % Divide up every sample

net.divideParam.trainRatio = 70/100;

net.divideParam.valRatio = 15/100;

net.divideParam.testRatio = 15/100;

% Choose a Performance Function

% For a list of all performance functions type: help nnperformance

net.performFcn = 'crossentropy'; % Cross-Entropy

% Choose Plot Functions

% For a list of all plot functions type: help nnplot

net.plotFcns = {'plotperform','plottrainstate','ploterrhist', ...

'plotconfusion', 'plotroc'};

% Train the Network

[net,tr] = train(net,x,t);

% Test the Network

y = net(x);

e = gsubtract(t,y);

performance = perform(net,t,y)

tind = vec2ind(t);

yind = vec2ind(y);

percentErrors = sum(tind ~= yind)/numel(tind);

% Recalculate Training, Validation and Test Performance

trainTargets = t .* tr.trainMask{1};

valTargets = t .* tr.valMask{1};

testTargets = t .* tr.testMask{1};

trainPerformance = perform(net,trainTargets,y)

valPerformance = perform(net,valTargets,y)

testPerformance = perform(net,testTargets,y)

% View the Network

view(net)

% Plots

% Uncomment these lines to enable various plots.

%figure, plotperform(tr)

%figure, plottrainstate(tr)

%figure, ploterrhist(e)

%figure, plotconfusion(t,y)

%figure, plotroc(t,y)

% Deployment

% Change the (false) values to (true) to enable the following code blocks.

% See the help for each generation function for more information.

if (false)

% Generate MATLAB function for neural network for application

% deployment in MATLAB scripts or with MATLAB Compiler and Builder

% tools, or simply to examine the calculations your trained neural

% network performs.

genFunction(net,'myNeuralNetworkFunction');

y = myNeuralNetworkFunction(x);

end

if (false)

% Generate a matrix-only MATLAB function for neural network code

% generation with MATLAB Coder tools.

genFunction(net,'myNeuralNetworkFunction','MatrixOnly','yes');

y = myNeuralNetworkFunction(x);

end

if (false)

% Generate a Simulink diagram for simulation or deployment with.

% Simulink Coder tools.

gensim(net);

end

## References

[1] Aaronson N.K., Mattioli V., Minton O., Weis J., Johansen C., Dalton S.O., Leeuw I.M.V.-D., Stein K.D., Alfano C.M., Mehnert A. (2014). Beyond treatment—Psychosocial and behavioural issues in cancer survivorship research and practice. Eur. J. Cancer Suppl..

[2] National Comprehensive Cancer Network (2019). NCCN Clinical Practice Guidelines in Oncology: Distress Management.

[3] Zabora J., Brintzenhofe Szoc K., Curbow B., Hooker C., Piantadosi S. (2001). The prevalence of psychological distress by cancer site. J. Psychol. Soc. Behav. Dimens. Cancer.

[4] Mehnert A., Hartung T.J., Friedrich M., Vehling S., Brähler E., Härter M., Keller M., Schulz H., Wegscheider K., Weis J. (2017). One in two cancer patients is significantly distressed: Prevalence and indicators of distress. Sycho Oncol. J. Psychol. Soc. Behav. Dimens. Cancer.

[5] Wang G.-L., Cheng C.-T., Feng A.-C., Hsu S.-H., Hou Y.-C., Chiu C.-Y. (2017). Prevalence, risk factors, and the desire for help of distressed newly diagnosed cancer patients: A large-sample study. Palliat. Support. Care.

[6] Skarstein J., Aass N., Fosså S.D., Skovlund E., Dahl A.A. (2000). Anxiety and depression in cancer patients: Relation between the Hospital Anxiety and Depression Scale and the European Organization for Research and Treatment of Cancer Core Quality of Life Questionnaire. J. Psychosom. Res..

[7] Schiel R.O., Brechtel A., Hartmann M., Taubert A., Walther J., Wiskemann J., Rötzer I., Becker N., Jäger D., Herzog W. (2014). Multidisciplinary health care needs of psychologically distressed cancer patients in a Comprehensive Cancer Center. Dtsch. Med. Wochenschr..

[8] von Essen L., Larsson G., Oberg K., Sjödén P.O. (2002). ‘Satisfaction with care’: Associations with health-related quality of life and psychosocial function among Swedish patients with endocrine gastrointestinal tumours. Eur. J. Cancer Care.

[9] Holland R.R., Ellis C.A., Geller B.M., Plante D.A., Secker-Walker R.H. (1999). Life expectancy estimation with breast cancer: Bias of the declining exponential function and an alternative ot its use. J. Clin. Psychol. Med. Settings.

[10] Kennard B.D., Stewart S.M., Olvera R., Bawdon R.E., Hailin A.O., Lewis C.P., Winick N.J. (2004). Nonadherence in Adolescent Oncology Patients: Preliminary Data on Psychological Risk Factors and Relationships to Outcome. J. Clin. Psychol. Med. Settings.

[11] Yee M.K., Sereika S.M., Bender C.M., Brufsky A.M., Connolly M.C., Rosenzweig M. (2017). Symptom incidence, distress, cancer-related distress, and adherence to chemotherapy among African American women with breast cancer. Cancer.

[12] Carlsen K., Dalton S.O., Diderichsen F., Johansen C. (2008). Risk for unemployment of cancer survivors: A Danish cohort study. Eur. J. Cancer.

[13] Carlson L.E., Walker A., Mitchell A.J. (2012). Screening for distress and unmet needs in patients witch cancer: Review and recommendations. J. Clin. Oncol..

[14] Mitchell A.J. (2013). Screening for cancer-related distress: When is implementation successful and when is it unsuccessful?. Acta Oncol..

[15] Hart S.L., Hoyt M.A., Diefenbach M., Anderson D.R., Kilbourn K.M., Craft L.L., Steel J.L., Cuijpers P., Mohr D., Berendsen M. (2012). Meta-Analysis of Efficacy of Interventions for Elevated Depressive Symptoms in Adults Diagnosed with Cancer. JNCI J. Natl. Cancer Inst..

[16] Faller H., Schuler M., Richard M., Heckl U., Weis J., Küffner R. (2013). Effects of Psycho-Oncologic Interventions on Emotional Distress and Quality of Life in Adult Patients with Cancer: Systematic Review and Meta-Analysis. J. Clin. Oncol..

[17] Arving C., Brandberg Y., Feldman I., Johansson B., Glimelius B. (2014). Cost-utility analysis of individual psychosocial support interventions for breast cancer patients in a randomized controlled study. Psycho-Oncology.

[18] Riedl D., Gastl R., Gamper E., Arnold C.R., Dejaco D., Schoellmann F., Rumpold G. (2018). Cancer patients’ wish for psychological support during outpatient radiation therapy. Strahlenther. Onkol..

[19] Holland J.C. (2018). Psycho-oncology: Overview, obstacles and opportunities. Psycho-Oncology.

[20] Suurmeijer T.P., Doeglas D.M., Briançon S., Krijnen W.P., Krol B., Sanderman R., Moum T., Bjelle A., Heuvel W.J.V.D. (1995). The measurement of social support in the ‘European research on incapacitating diseases and social support’: The development of the Social Support Questionnaire for Transactions (SSQT). Soc. Sci. Med..

[21] Sebri V., Mazzoni D., Triberti S., Pravettoni G. (2021). The Impact of Unsupportive Social Support on the Injured Self in Breast Cancer Patients. Front. Psychol..

[22] Sebri V., Triberti S., Pravettoni G. (2020). Injured Self: Autobiographical Memory, Self-Concept, and Mental Health Risk in Breast Cancer Survivors. Front. Psychol..

[23] Morel N., Dayan J., Piolino P., Viard A., Allouache D., Noal S., Levy C., Joly F., Eustache F., Giffard B. (2015). Emotional specificities of autobiographical memory after breast cancer diagnosis. Conscious. Cogn..

[24] McGinty H.L., Small B.J., Laronga C., Jacobsen P.B. (2016). Predictors and patterns of fear of cancer recurrence in breast cancer survivors. Health Psychol..

[25] Carlson L.E., Angen M., Cullum J., Goodey E., Koopmans J., Lamont L., Macrae J.H., Martin M., Pelletier G., Robinson J. (2004). High levels of untreated distress and fatigue in cancer patients. Br. J. Cancer.

[26] Verdonck-de Leeuw I.M., de Bree R., Keizer A.L., Houffelaar T., Cuijpers P., van der Linden M.H., Leemans C.R. (2009). Computerized prospective screening for high levels of emotional distress in head and neck cancer patients and referral rate to psychosocial care. Oral Oncol..

[27] Loth F.L., Meraner V., Holzner B., Singer S., Virgolini I., Gamper E.M. (2018). Following patient pathways to psycho-oncological care: Identification of treatment needs by clinical staff and electronic screening. Psycho-Oncology.

[28] Günther M.P., Kirchebner J., Ben Schulze J., Götz A., von Känel R., Euler S. (2022). Uncovering Barriers to Screening for Distress in Patients with Cancer via Machine Learning. J. Acad. Consult. Psychiatry.

[29] Günther M.P., Schulze J.B., Jellestad L., Mehnert-Theuerkauf A., von Känel R., Euler S. (2021). Mental disorders, length of hospitalization, and psychopharmacy—New approaches to identify barriers to psychological support for patients with cancer. Psycho-Oncology.

[30] Knies A.K., Jutagir D.R., Ercolano E., Pasacreta N., Lazenby M., McCorkle R. (2019). Barriers and facilitators to implementing the commission on cancer's distress screening program standard. Palliat. Support. Care.

[31] Fradgley E.A., Byrnes E., McCarter K., Rankin N., Britton B., Clover K., Carter G., Bellamy D., Paul C.L. (2020). A cross-sectional audit of current practices and areas for improvement of distress screening and management in Australian cancer services: Is there a will and a way to improve?. Support Care Cancer.

[32] Schweer C., Doering S., Haier J., Heuft G., Fritz F., Dugas M., Schneider G. (2011). Psychooncological interventions—What do cancer patients aged 60 years or older wish for?. Z. Psychosom. Med. Psychother..

[33] Ernst J., Zenger M., Schmidt R., Schwarz R., Brähler E. (2010). Medical and psychosocial care needs of cancer patients: A systematic review comparing urban and rural provisions. Dtsch. Med. Wochenschr..

[34] Dee E.C., Nipp R.D., Muralidhar V., Yu Z., Butler S.S., Mahal B.A., Nguyen P.L., Sanford N.N. (2021). Financial worry and psychological distress among cancer survivors in the United States, 2013—2018. Support. Care Cancer.

[35] Kriegeskorte N., Golan T. (2019). Neural network models and deep learning. Curr. Biol..

[36] Aruna P., Puviarasan N., Palaniappan B. (2005). An investigation of neurofuzzy system in psychosomatic dirsorders. Exp. Syst. Appl..

[37] Zou Y., Shen Y., Shu L., Wang Y., Feng F., Xu K., Qu Y., Song Y., Zhong Y., Wang M. (1996). Artificial Neural Network to Assist Psychiatric Diagnosis. Br. J. Psychiatry.

[38] Chattopadhyay S., Kaur P., Rabhi F., Acharya U.R. (2011). Neural Network Approaches to Grade Adult Depression. J. Med. Syst..

[39] Fan R., Hua T., Shen T., Jiao Z., Yue Q., Chen B., Xu Z. (2021). Identifying patients with major depressive disorder based on tryptophan hydroxylase-2 methylation using machine learning algorithms. Psychiatry Res..

[40] Davis G., Lowell W.E. (1993). A neural network that predicts psychiatric length of stay. MD. Comput. Comput. Med. Pract..

[41] Lyu J., Zhan J. (2019). BP neural network prediction model for suicide attempt among Chinese rural residents. J. Affect. Disord..

[42] Huan J., Chen J. (2011). BP neural networrk model for early diagnosis of Kawasaki disease. Biomed. Eng. Res..

[43] Ownby K.K. (2019). Use of the Distress Thermometer in Clinical Practice. J. Adv. Pract. Oncol..

[44] Holland J.C., Bultz B.D. (2007). The NCNN guideline for distress management: A case for making distress the sixth vital sign. J. Natl. Compr. Cancer Netw..

[45] Bertero L., Massa F., Metovic J., Zanetti R., Castellano I., Ricardi U., Papotti M., Cassoni P. (2018). Eighth edition of the UICC classification of malignant tumours: An overview of the changes in the pathological TNM classification criteria—What has changed and why?. Virchows Arch..

[46] Charlson M.E., Pompei P., Ales K.L., MacKenzie C.R. (1987). A new method of classifying prognostic comorbidity in longitudinal studies: Development and validation. J. Chronic Dis..

[47] Quan H., Li B., Couris C.M., Fushimi K., Graham P., Hider P., Januel J.-M., Sundararajan V. (2011). Updating and Validating the Charlson Comorbidity Index and Score for Risk Adjustment in Hospital Discharge Abstracts Using Data From 6 Countries. Am. J. Epidemiol..

[48] Kim D.H., Park H.C., Cho A., Kim J., Yun K.-S., Kim J., Lee Y.-K. (2021). Age-adjusted Charlson comorbidity index score is the best predictor for severe clinical outcome in the hospitalized patients with COVID-19 infection. Medicine.

[49] Kutner M.H. (2005). Applied Linear Statistical Models.

[50] de Vries A., Sollner W., Steixner E., Auer V., Schiessling G., Stzankay A., Iglseder W., Lukas P. (1998). Subjective psychological stress and need for psychosocial support in cancer patients during radiotherapy treatment. Strahlenther Onkol..

[51] Faller H., Olshausen B., Flentje M. (2003). Emotional distress and needs for psychosocial support among breast cancer patients at start of radiotherapy. Psychother. Psychosom. Med. Psychol..

[52] Carolan C., Smith A., Davies G., Forbat L. (2017). Seeking, accepting and declining help for emotional distress in cancer: A systematic review and thematic synthesis of qualitative evidence. Eur. J. Cancer Care.

[53] Clover K.A., Mitchell A.J., Britton B., Carter G. (2015). Why do oncology outpatients who report emotional distress decline help?. Psycho-Oncology.

[54] Kacel E.L., Pereira D.B., Estores I.M. (2019). Advancing supportive oncology care via collaboration between psycho-oncology and integrative medicine. Support Care Cancer.

[55] Stark A., Kaduszkiewicz H., Stein J., Maier W., Heser K., Weyerer S., Werle J., Wiese B., Mamone S., König H.-H. (2018). A qualitative study on older primary care patients’ perspectives on depression and its treatments—Potential barriers to and opportunities for managing depression. BMC Fam. Pract..

[56] Latalova K., Kamaradova D., Prasko J. (2014). Perpectives on perceived stigma and self-stigma in adult male patients with depression. Neuropsychiatr. Dis. Treat..

[57] Bracke P., Delaruelle K., Verhaeghe M. (2019). Dominant Cultural and Personal Stigma Beliefs and the Utilization of Mental Health Services: A Cross-National Comparison. Front. Sociol..

[58] Dilworth S., Higgins I., Parker V., Kelly B., Turner J. (2014). Patient and health professional’s perceived barriers to the delivery of psychosocial care to adults with cancer: A systematic review. Psycho-Oncology.

[59] Pichler T., Dinkel A., Marten-Mittag B., Hermelink K., Telzerow E., Ackermann U., Belka C., Combs S.E., Gratzke C., Gschwend J. (2019). Factors associated with the decline of psychological support in hospitalized patients with cancer. Psycho-Oncology.

[60] Frey Nascimento A., Tondorf T., Rothschild S.I., Koller M.T., Rochlitz C., Kiss A., Schaefert R.M., Meinlschmidt G.P., Hunziker S., Gaab J. (2019). Oncologist recommendation matters—Predictors of psycho-oncological service uptake in oncology outpatients. Psycho-Oncology.

[61] Ben Schulze J., Günther M.P., Riemenschnitter C., Wicki A., von Känel R., Euler S. (2021). Distinct psycho-oncological support inclinations and needs in patients with cancer: A large sample latent class analysis approach. Gen. Hosp. Psychiatry.

[62] Jacobsen Paul B., Donovan Kristine A., Trask Peter C., Fleishman Stewart B., Zabora J., Baker F., Holland J.C. (2005). Screening for psychologic distress in ambulatory cancer patients. Cancer..

[63] Shim E.-J., Shin Y.-W., Jeon H.J., Hahm B.-J. (2008). Distress and its correlates in Korean cancer patients: Pilot use of the distress thermometer and the problem list. Psycho-Oncology.

[64] Hegel Mark T., Collins E.D., Kearing S., Gillock K.L., Moore C.P., Ahles T.A. (2008). Sensitivity and specificity of the Distress. Thermometer for depression in newly diagnosed breast cancer patients. Psycho-Oncology.

[65] Acquati C., Kayser K. (2017). Predictors of psychological distress among cancer patients receiving care at a safety-net institution: The role of younger age and psychosocial problems. Support. Care Cancer.

[66] Günther M.P., Schulze J.B., Kirchebner J., Jordan K.-D., von Känel R., Euler S. (2022). Severe mental illness in cancer is assosciated with disparities in psycho-oncological support. Curr. Probl. Cancer.

[67] Guy G.P., Machlin S.R., Ekwueme D.U., Yabroff K.R. (2015). Prevalence and costs of skin cancer treatment in the U.S.; 2002–2006 and 2007–2011. Am. J. Prev. Med..

[68] Wiggins B., Corsini N., Ramsey I., Edwards S., Ball D., Cocks L., Lill J., Sharplin G., Wilson C. (2018). An evaluation of social work services in a cancer accomodation facility for rural South Australians. Support Cancer Care.

[69] Hyer J.M., Paredes A.Z., Palmer Kelley E., Tsilimigras D., Meyer B., Newberry H., Pawlik T.M. (2021). Characterizing pastoral care utilization by cancer patients. Am. J. Hosp. Palliat. Care.

[70] Palmer Kelly E., Hyer J.M., Paredes A.Z., Tsilimigras D., Meyer B., Newberry H., Pawlik T.M. (2021). Provision of supportive spiritual care for hepatopancreatic cancer patients: An unmet need?. HPB.

[71] Moghimian M., Irajpour A. (2019). The requirements of hospital-based spiritual care for cancer patients. Support. Care Cancer.

[72] Cutillo A., O’Hea E., Person S.D., Lessard D., Harralson T.L., Boudreaux E. (2017). The Distress Thermometer: Cutoff Points and Clinical Use. Oncol. Nurs. Forum.

[73] Ploos van Amstel F., Tol J., Sessink K., van der Graaf W.T., Prins J., Ottevanger P. (2017). A specific distress cutoff score shortly after breast cancer diagnosis. Cancer Nurs..

[74] Zwahlen D., Tondorf T., Rothschild S., Koller M.T., Rochlitz C., Kiss A. (2017). Understanding why cancer patients accept or turn down psycho-oncological support: A prospective observational study including patients’ and clinicians’ perspectives on communication about distress. BMC Cancer.

